# Introduction of Auricular Acupuncture in Elderly Patients Suffering from Major Depression: Protocol of a Mixed Methods Feasibility Study

**DOI:** 10.1155/2015/678410

**Published:** 2015-04-15

**Authors:** Janina Geib, Monika A. Rieger, Stefanie Joos, Gerhard W. Eschweiler, Thomas Dresler, Florian G. Metzger

**Affiliations:** ^1^Department of Psychiatry and Psychotherapy, University Hospital Tübingen, Calwerstraße 14, 72076 Tübingen, Germany; ^2^Geriatric Center at the University Hospital Tübingen, Calwerstraße 14, 72076 Tübingen, Germany; ^3^Institute for Occupational and Social Medicine, and Health Services Research, University of Tübingen, Wilhelmstraße 27, 72074 Tübingen, Germany; ^4^Coordination Centre, Core Facility for Health Services Research, Faculty of Medicine, Eberhard Karls University of Tübingen, Wilhelmstraße 27, 72074 Tübingen, Germany; ^5^Department of General Practice, University of Tübingen, Österbergstraße 9, 72074 Tübingen, Germany; ^6^LEAD Graduate School, University of Tübingen, Gartenstraße 29a, 72074 Tübingen, Germany

## Abstract

*Background*. Due to an increasing number of elderly people suffering from major depression and potential side effects of the prescribed drugs, the introduction of new therapeutic approaches is needed. Currently, in Germany, auricular acupuncture is no part of clinical care for gerontopsychiatric patients. Based on promising clinical experiences and existing evidence for treating addiction and trauma, a benefit of auricular acupuncture integrated in existing treatment programs in elderly patients may be hypothesized. Within this project auricular acupuncture according to the National Acupuncture Detoxification Association (NADA) will be integrated in the multimodal treatment regime for elderly patients with major depression in a daytime ward setting. *Methods/Design*. To evaluate the feasibility and acceptance a mixed method approach is used. In a day clinic, a sample of 20 psychogeriatric patients with the diagnosis of major depression will be enrolled. The patients will receive a total of nine auricular acupuncture treatments according to the standardized NADA protocol in a group setting. The therapeutic process, its organization, the experience, and the willingness of patients to participate will be evaluated by interviews with patients and the therapeutic team. Data will be analyzed qualitatively using content analysis. Additionally, quantitative outcome parameters will be measured by standardized questionnaires.

## 1. Background

In recent decades, acupuncture has become increasingly important in the western medicine and also in Germany and receives great popularity among patients.

According to evidence based medicine (EBM) acupuncture is at the level of evidence 1A in treating postoperative dental pain as well as nausea and vomiting [1]. Clear positive evidence for the efficacy exists, according to Stör and Irnich [1], in headaches, low back pain, temporomandibular dysfunction, fibromyalgia and symptoms of osteoarthritis of the knee, and epicondylitis. Regarding postoperative pain, a meta-analysis of 15 randomized clinical trials (RCTs) showed a significant reduction of pain after surgery and a reduction of the cumulative opioid dosage of 30% and, therefore, fewer side effects by opioids [2]. Furthermore, a significant anxiolysis before surgery could be demonstrated in two ear acupuncture studies [3, 4].

Auricular acupuncture is a special form of acupuncture which was first described in 2500 B.C. Since 1950 auricular acupuncture was introduced to Europe by the French Paul Nogier. In 1985, a protocol for treatment of addictions was developed in New York. In Germany, the NADA (National Acupuncture Detoxification Association) society was founded in 1993. According to the NADA protocol thin disposable steel needles are inserted at 3–5 points (= point areas) at both ears at a depth of 2-3 mm: autonomic, shen men, kidney, liver, and lung [5]. An essential difference of the NADA protocol in contrast to conventional acupuncture represents the group setting: patients are offered auricular acupuncture sessions at fixed time schedules in a group therapy at a high frequency (up to daily). The acceptance of passive participation in the group and, thus, the nonconfrontative design are main characteristics. Even special trained medical staff members are allowed to perform auricular acupuncture [5]. Auricular acupuncture is particularly suited for psychiatric patients, as the patient does not have to undress and can assume a sitting position, similar to psychotherapy. The patient remains at eye level with the therapist without being in an inferior and passive position by having to lie down undressed.

The application of auricular acupuncture in Germany is increasing; in 2004, 230 outpatient and inpatient facilities applied the protocol for the treatment of addictive disorders, but also for other mental disorders [5]. In 2009, 30,000 trained therapists in Germany already used this form of acupuncture at least occasionally [1].

Numerous studies found positive clinical effects for the treatment with auricular acupuncture for different indications in substance dependency [6, 7], though controversial aspects exist. So far only small randomized controlled trials have been conducted. Recently, the application of auricular acupuncture according to the NADA protocol has been further expanded: in a recent study, traumatized refugees in Kenya were stabilized mentally by this treatment [8]. 130 prisons in England use this protocol and showed a decrease of physical assaults up to 80% [9]. A positive correlation between the successful completion of the therapy-programme of patients with cooccurring borderline personality disorder and nicotine dependence was found for the additional use of “acudetox” [10]. A relaxing, concentration enhancing, internally stabilizing, anxiety reducing, and sleep-regulating effect of acupuncture on the NADA protocol has been described several times [11] and gives impact for further psychiatric and psychosomatic indications [12].

Auricular acupuncture may contribute to reaching this aim whereas ear acupuncture is used in addition to medication in the present design. However, two of 30 studies in progress treating major depression with (body) acupuncture showed positive effects of a combination of acupuncture and pharmacotherapy as compared to only pharmacotherapy [13]. Particularly restlessness is a goal of auricular acupuncture, so Payer et al. [14] assume that the auricular acupuncture for (nonaddicted) psychiatric patients promotes inner self-healing and regulates disturbances in well-being. A relaxing effect in case of excessive tension, an increase of concentration, wakefulness in permanent fatigue, and an improvement of nocturnal sleep in case of sleep disorders have been repeatedly demonstrated yet [15, 16].

Since there is little experience with auricular acupuncture in psychogeriatrics, the study adopts an implementation approach with mixed methods design [17]. It combines a qualitative part with interviews of the participants (before and after intervention) and the therapeutic team (before and after intervention) and a quantitative part with neurophysiological assessments (before and after intervention) in order to answer the following questions.

*Main Research Questions*

- Is it feasible to integrate auricular acupuncture in a multimodal pharmacological and psychotherapeutic treatment regime of the elderly with major depression in a day clinic setting?
- Do the depressed elderly patients accept auricular acupuncture? If rejected, what are the reasons?
- What are barriers and enablers for acceptance of acupuncture treatment?
- Is it feasible to integrate auricular acupuncture in the therapeutic team?

*Further Research Questions*

- Is the setting suitable with 45 minutes three times weekly for three weeks?
- How are the effects of the auricular acupuncture assessed (positive, useful, or even harmful) by the patient and the therapeutic team?
- Are the questionnaires used for neuropsychological assessment feasible?

## 2. Methods

### 2.1. Participants

As a first step it is planned to recruit 20 patients (>60 years old) suffering from a major depression episode (ICD-10: F32-F33) via the psychogeriatric day clinic of the Department of Psychiatry and Psychotherapy, University Hospital of Tübingen (participating patients). Excluding criteria are severe internistic or neurological diseases, inflammation, skin ulcerations, or wounds in the ear, pronounced tendency to collapse, and psychiatric (co)morbidity such as schizophrenia or (psychomotoric) restlessness that prevents sitting still for 45 minutes. Patients need to be able to give informed consent. Mild cognitive impairment or mild dementia is not an exclusion criterion.

A second group will consist of patients who refuse a participation in acupuncture but agree to giving an interview (refusing patients). They will receive treatment as usual with psychotherapy, psychotropic medication, and occupational and physical therapy. Furthermore, the nursing staff of the day clinic will be interviewed.

The study was reviewed and approved by the Ethics Committee of the Medical Faculty and University Hospital of Tübingen (project number 330/2013BO1), and all procedures involved are in accordance with the latest version of the Declaration of Helsinki. All studied subjects have to give a written informed consent.

### 2.2. Intervention

**Figure 1:**
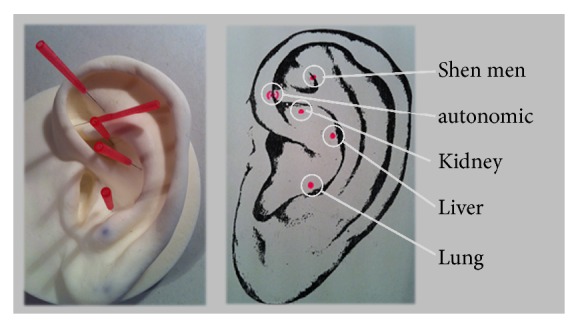
NADA auricular acupuncture.

Auricular acupuncture sessions will take place three times per week for a period of 30 to 45 minutes before lunch. The individual observation period is a total of three weeks. The participants are sitting in a quiet room on a chair, take a comfortable position, and are required to sit still and be quiet. The 3–5 point areas (autonomic, shen men, kidney, liver, and lung; see Figure 1: NADA auricular acupuncture) are pierced on both ears with a thin disposable steel needle 2-3 mm deep by a trained physician or nurse.

#### 2.2.1. Qualitative Research Methods and Selected Items

We will use a mixed method design to answer the research questions. As qualitative data collection methods interviews with patients and group interviews of the therapeutic team will be used.

To answer the research questions, we will perform semistandardized interviews with patients before and after acupuncture treatment as follows.

1. The patients participating in the acupuncture will be interviewed in individual semistructured interviews before and after the 3-week period of acupuncture treatment.
2. The therapeutic staff will have focus groups at 2 time points: at the beginning and after the end of the study.

The individual and group interviews will be digitally recorded and transcribed. These will be analyzed using special software (e.g., MaxQDA) according to the rules of qualitative content analysis as in the work of Mayring [18].

An interview guideline has been developed: patients will be asked about expectation and ideas to acupuncture and openness towards novelty. After the end of treatment questions concerning the subjective satisfaction with and effectiveness of the applied method will be asked. At the beginning information about former experience with acupuncture treatments and traditional Chinese medicine will be collected by a standardized questionnaire as well as age, diagnosis, duration of disease, and pharmaceutical use.

Patients who refuse their participation will be asked about the reasons for refusing acupuncture, potential doubts, and possible negative experiences with acupuncture.

The therapeutic staff in psychogeriatric units typically comprises several professions, consisting of physicians (psychiatrists, neurologists, or interns), psychological psychotherapists, geriatric nurses and trainees, occupational therapists, physiotherapists, and social workers. In problem-focused group interviews (before and after intervention), the perceived usefulness of the treatment, the amount of human effort, and the results for the individual patient will be discussed among all involved staff members.

Additionally, objective parameters will be measured to complement process evaluation: the duration of acupuncture, the individual active and passive participation, and the total number of patients per acupuncture appointment will be documented.

#### 2.2.2. Quantitative  Research Methods: Neuropsychological Testing

Before the treatment and at the end, the following instruments will be applied to the patients. These instruments are part of the routine diagnostic inpatient treatment concept.

To assess depressive symptoms, the Geriatric Depression Scale (GDS; self-rating scale; 10) and the Hamilton Depression Scale (HAM-D; external assessment scale; 11) will be used. For evaluation of cognitive function and orientation, the Mini-Mental State Examination (MMSE, 12) will be used. The current quality of life will be measured with the SF-36 [19]. For information about the quality of sleep the Pittsburgh Sleep Quality Index (PSQI; 22) will be used. The collected data of standardized instruments will be quantitatively analyzed by repeated measures ANOVAs and *t*-test for paired samples. Nonparametric tests will be applied whenever necessary.

*Geriatric Depression Scale (GDS)*. The GDS is a self-report assessment for older patients consisting of 30 items. Items require “yes” or “no” statements, so that mild cognitive impaired (MCI) patients are also able to fill in this questionnaire. The highest possible score is 30 points. Severe depression is indicated by scores of 20–30, mild depression by scores of 10–19, and a normal score by scores below 9 [20].

*Hamilton Depression Scale (HAM-D)*. The HAM-D is an observer-rating questionnaire with 17 items to describe the severity of cognitive and bodily symptoms of a major depression. Each item is rated in 3- and 5-point scales. Paranoid symptoms and diurnal variation are not included in the scale, but they give further information about the depression [21].

For the HAM-D, no generally accepted cut-off scores are available. Yet, according to the current German clinical practice guideline on unipolar depression [22], some cutoffs provide practically relevant information.

Severe depression is indicated by scores over 24 points, moderate depression by scores of 15–24, mild depression by scores of 9–16, and a normal score by scores below 8.

*Mini-Mental State Examination (MMSE)*. The MMSE is an observer-rating questionnaire to screen for cognitive impairment. It includes orientation regarding time (5 points) and regarding location (5 points), registration (3 points), attention and calculation (5 points), recall (3 points), language (2 points), repetition (1 point), and complex commands (6 points) with a maximum of 30 points [23].

*Health-Related Quality of Life (SF-36)*. The SF-36 questionnaire measures the health-related quality of life by 36 questions. There are 8 scaled scores for the dimensions “physical functioning,” “role limitations because of physical health problems,” “bodily pain,” “social functioning,” “general mental health,” “role limitations because of emotional problems,” “vitality,” and “general health perceptions.” The highest possible score in each dimension is 100 (good health/no disability), and the lowest is 0 (poor health/maximum of disability) [19].

*Pittsburgh Sleep Quality Index (PSQI)*. The PSQI is a self-report assessment asking for the quality of sleep over a one-month interval. It consists of 19 items and 7 “component” scores: subjective sleep quality, sleep latency, sleep duration, habitual sleep efficiency, sleep disturbances, use of sleeping medication, and daytime dysfunction. The sum of the scores yields a total score (0–21 points). A total score over 5 points indicates poor sleep quality, a score under 5 points good sleep quality [24].

**Figure 2:**
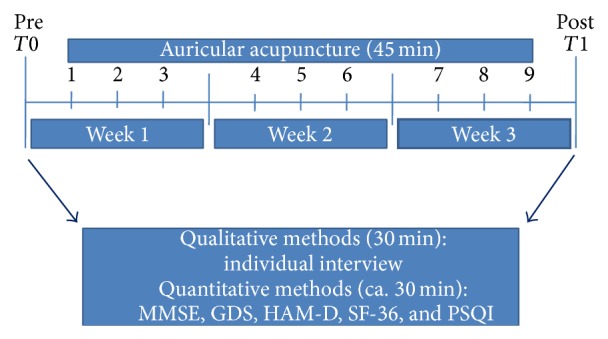
Time line.

An overview of the whole procedure is given in Figure 2: timeline.

## 3. Discussion

The described study design is investigating the feasibility of treating elderly depressive patients with auricular acupuncture. According to the feasibility design of the study a mixed methods approach is chosen. The main part is a qualitative design with focus on feasibility and acceptance to improve procedures. Additionally, quantitative data will be analyzed to get information about the acceptance of instruments and to get an impression about effect sizes and the differences between participating and refusing patients.

Since the efficacy of acupuncture in elderly depressive patients is not yet proven, such a project could be considered premature. A reason may be that psychogeriatric care is more complex than therapy of addiction due to the somatic comorbidity of elderly patients. This might be especially the case when side effects of pharmacologic therapies get more severe and the target effect decreases.

In addition, the elderly much more often reject innovations. Clinical experiences and existing evidence for NADA auricular acupuncture in addiction and comorbid conditions have led us to the idea of the implementation of the NADA protocol in psychogeriatric patients and to evaluate the process of the implementation first.

However, given reserved attitudes of elderly to new experiences, particularly body-experience, an introduction in psychogeriatrics may be regarded a demanding task although in case of demonstrated positive effects. Therefore, starting with a feasibility study also in terms of process factors is a reasonable approach.

### 3.1. Strengths and Limitations

The main strength of the study is the novelty of integrating acupuncture in a psychogeriatric treatment regimen. Another strength of the study is the involvement of the whole therapeutic team of the daytime clinic: according to the basis of NADA not only physicians and psychotherapists but also other health professionals such as nurses or occupational therapists are involved in the treatment itself. The multiprofessional therapeutic team is part of the implementation process and therefore is interviewed in focus groups. This is favourable for the introduction of a new therapy module, for this daytime clinic and for psychogeriatrics in general.

A disadvantage of the explorative design is the lack of a real control group: the second group consists of day-clinic patients rejecting auricular acupuncture and, hence, is not matched in age or gender. If wanted these patients are allowed to participate passively in the group setting. The criteria of a controlled study design are not fulfilled, so the quantitative results will be limited. Generally, the concept of auricular acupuncture is hardly suitable for sham-controlled designs. In particular, sham acupuncture in case of auricular acupuncture is difficult as acupuncture areas and no distinct acupuncture points are used. Therefore, a sham-electroacupuncture, in which the acupuncture-pen is switched off, could be used. Furthermore, the effect in psychiatric patients is not solely based on the acupuncture procedure but also on the setting with a group therapy and openness to an active and passive participation. However, our study will provide important information to plan a randomized-controlled confirmative study.

### 3.2. Relevance of the Study

Due to the increased life expectancy and the needs of older people with mental illness which will likely continue to increase, it is important to identify effective therapies and to seek innovative ways. Particularly in the area of major depressive disorders new treatments and therapeutic approaches are urgently needed. Many psychogeriatric patients are treated insufficiently over years, maybe marked as chronic or refractory depression, indicating a high level of suffering. Typical symptoms of these patients are loss of memory, insomnia, and restlessness. Psychotropic pharmacotherapy in the elderly includes a high risk of side effects, particularly in impaired renal or hepatic function. Additionally, multimorbidity often causes polypharmacy and interaction. Finally, nearly every psychotropic medication increases the risk of falls in the elderly [25, 26]. Therefore, alternative therapeutic options are urgently needed for a possible reduction of psychotropic drugs.

In case of positive results an aim of future studies might be the transfer of auricular acupuncture in an ambulatory setting addressing the reduction of hospital admissions by auricular acupuncture.
